# Establishment of a 4-miRNA Prognostic Model for Risk Stratification of Patients With Pancreatic Adenocarcinoma

**DOI:** 10.3389/fonc.2022.827259

**Published:** 2022-02-03

**Authors:** Xun Gong, Yuchen Liu, Chenglong Zheng, Peikai Tian, Minjie Peng, Yihang Pan, Xiaowu Li

**Affiliations:** ^1^ Department of Hepatobiliary Surgery, Shenzhen Key Laboratory, Guangdong Provincial Key Laboratory of Regional Immunity and Diseases, International Cancer Center, Shenzhen University General Hospital, Shenzhen University Clinical Medical Academy, Shenzhen University, Shenzhen, China; ^2^ College of Electronics and Information Engineering, Shenzhen University, Shenzhen, China; ^3^ Scientific Research Center, The Seventh Affiliated Hospital, Sun Yat-sen University, Shenzhen, China; ^4^ Big Data Center, The Seventh Affiliated Hospital, Sun Yat-sen University, Shenzhen, China

**Keywords:** miRNA, pancreatic adenocarcinoma, risk stratification, prognosis, treatment strategies

## Abstract

Pancreatic adenocarcinomas (PAADs) often remain undiagnosed until later stages, limiting treatment options and leading to poor survival. The lack of robust biomarkers complicates PAAD prognosis, and patient risk stratification remains a major challenge. To address this issue, we established a panel constructed by four miRNAs (miR-4444-2, miR-934, miR-1301 and miR-3655) based on The Cancer Genome Atlas (TCGA) and Human Cancer Metastasis Database (HCMDB) to predicted the prognosis of PAAD patients. Then, a risk prediction model of these four miRNAs was constructed by using Cox regression analysis with the least absolute shrinkage and selection operator (LASSO) regression analysis. This model stratified TCGA PAAD cohort into the low-risk and high-risk groups based on the panel-based risk score, which was significantly associated with 1-, 2-, 3-year OS (AUC=0.836, AUC=0.844, AUC=0.952, respectively). The nomogram was then established with a robust performance signature for predicting prognosis compared to clinical characteristics of pancreatic cancer (PC) patients, including age, gender and clinical stage. Moreover, two GSE data were validated the expressions of 4 miRNAs with prognosis/survival outcome in PC. In the external clinical sample validation, the high-risk group with the upregulated expressions of miR-934/miR-4444-2 and downregulated expressions of miR-1301/miR-3655 were indicated a poor prognosis. Furthermore, the cell counting kit-8 (CCK-8) assay, clone formation, transwell and wound healing assay also confirmed the promoting effect of miR-934/miR-4444-2 and the inhibiting effect of miR-1301/miR-3655 in PC cell proliferation and migration. Taken together, we identified a new 4-miRNA risk stratification model could be used in predicting prognosis in PAAD.

## Introduction

Pancreatic adenocarcinoma (PAAD) is one of the deadliest tumor types overall. Because PAAD-specific symptoms are not easy to detect in the early stage, they only appear in the advanced stage of the disease, resulting in an extremely low 5-year survival rate, only ~10% (1). The proportion of patients who have an opportunity to undergo surgical resection is less than 20% (2, 3). In addition, patients with PAAD who undergo complete tumor resection usually have local or distant recurrence within 2 years (4). Still, PAAD treatment remain a major challenge, with surgery being the only curative therapy available.

The poor prognosis of PAAD may be due to its invasion, resistance to treatment, and lack of early screening markers and diagnostic methods (5). Establishing a prognostic assessment model can provide guidance for the personalized treatment for high-risk pancreatic cancer patients to maximize survival time. Studies have shown that factors affecting prognosis include age, general health, comorbidities, and ability to tolerate treatment (6). Meanwhile, the prognosis evaluation system also includes some indicators that are commonly used to predict the condition of individual patients, such as histological grade, lymph node or distant metastasis, vascular and perivascular infiltration, and CA199 (7). However, due to the tumor heterogeneity of PAAD and the limited number of biomarkers available, so far there has been little clinical benefit (8, 9). Therefore, there is still a need for an assessment system with high sensitivity and specificity to predict the prognosis of PAAD patients. Molecular markers can be used to tailor personalized treatment strategies for patients with inoperable pancreatic cancer, which is particularly important for prognostic assessment (10).

With rapid development of next-generation sequencing technologies, most human cancers have now been comprehensively profiled, which has led to the recognition of previously unknown molecular subtypes, and thus improved prognosis, diagnosis, and tumor precision medicine. Although molecular subtyping is intriguing from a biological perspective, owing to each subtype being characterized by several hundred genes, and various molecular subtyping classification criteria for subtype(s) with poor prognosis have been introduced, the difficulty of performing transcriptome profiling for each patient, its translation into clinical practice has been challenging (11, 12).

Previous studies have convincingly demonstrated that microRNA (miRNA) expression is frequently dysregulated in malignancies, and a single miRNA can regulate the expression of dozens of target genes, with emerging evidence highlighting the potential of miRNAs as cancer biomarkers (13). Studies have shown that some miRNAs are better biomarkers than other known biomarkers, and have shown beneficial effects in treating diseases in preclinical studies (14). Taken together, a panel of prognostic miRNAs might offer a more robust and clinically meaningful signature for PAAD patient stratification and molecular subtyping.

In the current study, we screened publicly available datasets to identify PAAD metastasis-associated miRNAs. Following extensive bioinformatic analysis and biomarker validation, we established and validated a 4-miRNA panel for the prediction of prognosis by using TCGA PAAD and GEO cohorts. Then, the miRNAs signature for prediction of PAAD clinical outcomes was constructed using the least absolute shrinkage and selection operator (LASSO) regression and Cox regression analysis. We confirmed the risk score was an independent prognostic index of PAAD patients both in the training and testing cohorts. More importantly, PAAD patients were divided into the high- and low-risk groups based on the risk score successfully. In addition to these two risk groups possessing different PAAD prognoses, they also exhibited the expression profiles of high-risk group could also enhance the proliferation and migration of pancreatic cancer cells. Our research uncovered an underlying implication of 4 miRNAs-based signature, showing their potential as biomarkers for predicting clinical prognosis and therapy for PAAD patients.

## Materials and Methods

### Data Sourcing and Preprocessing and Clinical Information Distribution

During the biomarker discovery phase of the current study, TCGA datasets were analyzed for the identification of candidate miRNAs as pro-oncogene in patients with malignant metastasis PAAD. A total of 169 cases (metastatic n=28; non-metastatic n=141) with expression data were included, and gene expression profile was normalized by log2 for further analysis.

Among the 185 TCGA PAAD samples, 50% are randomly selected as the training cohort and 50% as the validation cohort. All samples are subjected to 2-fold cross validation with 1000 times repeats with “caret” package to ensure the stability of the modeling, and to ensure that the randomly selected samples and all samples in the distribution of clinical features is uniform. The distribution of clinical information (including age, survival status, gender, and TNM staging) of the training and validation cohorts were listed in **Supplementary Tables 1** and **2**, respectively.

### Analysis of Differential miRNA Expression Based on RNA-Seq Data

Use raw counts in TCGA RNA-seq and miRNA illuminaHiseq data for differential expression analysis. Use the edgeR package in R (15) for analysis, and adjust the degree of over-dispersion through the empirical Bayesian method. In this study, limma was used to calculate CPM, and only genes with CPM greater than 1 were considered. Use generalized linear models and likelihood ratio tests to determine significance and fold change (FC). When the adjusted p-value (by the Benjamini-Hochberg method) is less than 0.05 and the FC is greater than 1.5, the differential expression genes were screened as statistically significant. Through the above methods, we figured out the differential expression mRNA and miRNAs in metastatic and non-metastatic PAAD samples (16).

### Risk Model Construction

As the method described by Guo et al. (16). Lasso regression was used to construct a cox model from 11 differential miRNA. Most of the miRNA were eliminated because their coefficients are zero and leaving only miR-934, miR-4444-2, miR-1301 and miR-3655 for subsequent analysis of pancreatic cancer risk predictions.

We perform univariate and multivariate Cox regression analysis to generate and validated a 4-miRNA (miR-934, miR-4444-2, miR-1301 and miR-3655) risk prediction model in the validation cohort. Then, the PAAD patients in the training set and testing set were separated into the high-risk and low-risk groups according to the median risk score cutoff value.

### Functional Enrichment Analyses

To investigate relevant biological pathways in subjects of high- and low-risk PAAD groups. Gene Ontology (GO) and Kyoto Encyclopedia of Genes and Genomes (KEGG) pathway enrichment analyses were performed with “clusterprofiler” and visualized with “ggplot2” and “Goplot” packages in R (17–19). We performed Gene Set Enrichment Analysis (GSEA, 4.0.2), an enrichment map was generated with Cytoscape (20) (absolute NES>1, false discovery rate (FDR) < 0.05 adjusted by BH method).

### Nomogram Construction

Cox analyses were used to evaluate the prognostic ability of four miRNA signature in pancreatic cancer datasets, and their results were used to construct multiple nomograms with “rms” package in R (17). The discrimination of the model was determined based on concordance index (C-index) and calibration curves (*P*<0.05). In addition, Kaplan-Meier survival analysis was conducted to evaluate the difference of OS in the two groups. Meanwhile, the log-rank test was used to calculate statistical significance and *P* < 0.05 was considered significant. The clinical characteristics of the PAAD patients were combined with the prognostic of 4 miRNAs signature to construct a nomogram through the “rms” R package. Then, the accuracy and discriminative power of the nomogram were evaluated by drawing a calibration.

### Molecular Subtypes Classification of PAAD

There are different molecular subtype systems depicted previously (21–25), we retrieved original classification data and compared with our stratification group based on the 4-miRNA risk score. Sankey diagram were draw by use of R package ggalluvial.

### Immune Cell Infiltration Analysis and Cancer-Immunity Cycle

The corration of Immune cell type and risk group was according to the immune infiltrates scores based on CIBERSORT, XCELL, TIMER and MCPCOUNTER algorithms were retrieved from TIMER 2.0 database (http://timer.cistrome.org/). The Cell-type Identification by “CIBERSORT” R package was applied to estimate the fractions of 22 immune cell types in all PAAD patients (26).

### Validation of the Expression of the 4 miRNAs in Pancreatic Cell and GSE Database

At first, we examined the expressions of miR-6510 and miR-934 in two pancreatic cancer cell lines (Hs766t and Hs766t-L3) by qRT-PCR. RNA enriched for small non-coding RNAs was extracted from the cells using the miRNeasy Kit (Qiagen, Valencia, CA). The expression of miRNAs was quantified *via* TaqMan qRT-PCR assays (Applied Biosystems, Foster City, CA) on a QuantStudio 7 Flex Real-Time PCR System (Applied Biosystems). Primers for qRT-PCR of miR-6510 and miR-934 were as follows in **Supplementary Table 3**. U6 was used as endogenous controls for data normalization. 2^-ΔΔCT^ method was used to calculate the expression of each miRNA. Then, two GSE database GSE38781 (https://www.ncbi.nlm.nih.gov/geo/query/acc.cgi?acc=GSE38781) and GSE163031 (https://www.ncbi.nlm.nih.gov/geo/query/acc.cgi?acc=GSE163031) of pancreatic cancer were used to validate the influence of the expression of 4 miRNAs on the prognosis of pancreatic cancer.

### Fluorescence *In Situ* Hybridization

FISH assay was performed as previously described (27) on two pancreatic cancer tissue microarray slides (Cat# HPanA180Su03) that purchased from Outdo Biotech Co. (Shanghai, China). A total of 180 pancreatic cancer tissue samples with prognosis information were included. Specific probe targeting miR-934, miR-4444-2, miR-1301 and mi-R3655, namely, has-miR934 (5’- CCAGTGTCTCCAGTAGTAGACA-3’), has-miR4444-2 (5’- CGCCTCTTCCAACTCGAG-3’), has-miR1301 (5’-GAAGTCACTCCCAGGCAGCTGCAA-3’) and has-miR3655 (5’- AGCAACACCGCAGCGACAAGC-3’) was purchased from Servicebio Co. (Wuhan, China). Nuclei were stained with DAPI. All procedures were conducted according to the manufacturer’s instructions, and slides were scanned by NanoZoomer (Japan) followed by scoring based on the portion of positive signal cells.

### Cell Culture and the 4 miRNAs Overexpressed Transfected

The pancreatic cell line CFPAC was acquired from ATCC company (Manassas, VA, USA). Dulbecco’s modified Eagle’s medium (DMEM, Gibco, Carlsbad, CA, United States) supplemented with 10% fetal bovine serum (FBS, Gibco, Carlsbad, CA, United States) were utilized for the culture of CFPAC cells at 37°C in 5% CO_2_ atmosphere.

The 4 miRNA mimics were designed and synthesized by GenePharma (Shanghai, China). The miRNA mimics (**Supplementary Table 4**) were transfected into CFPAC cells using lipofectamine RNAiMAX reagent (Invitrogen, United States) according to the manufacturer’s protocols. Then, qRT-PCR was used to evaluate transfection efficiency after 24h.

### Cell Proliferation, Colony-Formation Assay, Transwell and Wound Healing Assay

The proliferation abilities of CFPAC cells transfected with 4 miRNAs were assessed using a cell counting kit-8 (CCK-8) assay (Yeasen, Shanghai, China) according to the manufacturer’s instructions. 10 μL CCK-8 reagent was directly added to each well of a 96-well plate that has overexpressed miRNA cells at the specified time (1, 2, 3, 4, and 5d), and then incubated at 37°C for 1.5 h. Finally, optional density (OD) was measured by a microplate reader (BioTek Instruments, United States) at 450 nm. For the colony-formation assay, freshly isolated cells were plated at clonal density (1,000 cells per well) in matrigel-coated 6-well dishes for 14 days, after which they were fixed, dyed, and counted.

A 24-well transwell migration chamber (8 μm pore size, Corning, USA) was used for transwell migration determination. The overexpressed miRNA cells were resuspended in 200 μL serum-free DMEM and seeded into the inner chamber. Add 500 μL of DMEM medium containing 20% FBS as an attractant to the bottom chamber. After 24 hours of incubation, cells that did not pass through the membrane were gently removed with cotton balls, and the cells that passed through the membrane were fixed with 4% paraformaldehyde and stained with crystal violet for 15 min. The number of migrated cells is calculated by Image J software.

Wound healing test was performed to detect the migration ability of cells after overexpression of miRNA. The miRNAs transfected cells were spread on a 6-well plate and grown to 90% confluence. Use a 200 μL micropipette tip to make a straight scratch on the single cell layer in each well, apply phosphate buffered saline (PBS) to wash away the shed cell debris, and then culture the cells in serum-free DMEM medium. The horizontal distance of the migrating cells was photographed by a microscope for 24 hours and measured by Image J software.

### Statistical Analysis

The Kaplan-Meier survival curve was drawn based on the critical value of survival rate between the high-risk group and the low-risk group. Univariate and multivariate Cox regression analysis were used to determine whether the identified biomarkers are independent prognostic factors. All analyses were performed using R version 3.6.3. Experiments in this study were performed in triplicate with the statistical results presented as means ± standard deviation (SD) using GraphPad Prism Software (version 9.3, CA, United States). Student t-test was applied to compare the differences between the two groups. Differences were considered statistically significant if the p-value was < 0.05.

## Results

### Identification of miR-934 Associated With Metastasis and Poor Prognosis in PAAD

**Figure 1:**
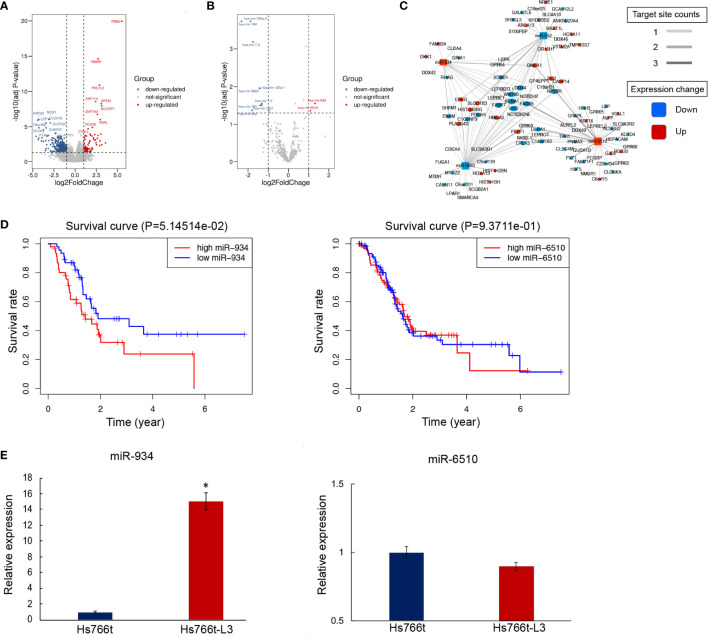
Differential mRNA and miRNA expression in pancreatic adenocarcinomas (PAAD) subjects with/out metastasis. **(A, B)** Overall pattern of differential mRNA gene **(A)** and miRNA **(B)** expression in PAAD with/out metastasis is presented *via* a volcano plot. **(C)** Net correlation of mRNA and miRNA. **(D)** Kaplan-Meier curves showing overall survival (OS) of PAAD patients stratified based on miR-934 expression levels. **(E)** Relative expression of miR-6510 and miR-934 in Hs766t and Hs766t-L3 cells (*t* test, *P < 0.05).

To investigate differential gene expression between PAAD with/without metastasis, we analyzed RNA-seq data from samples deposited in the TCGA data portal using the edgeR program, including 28 metastatic samples and 141 non-metastatic samples. The adjusted p-value and the FC cutoffs were set at 0.05 and 1.5, respectively. The number of genes upregulated in metastatic subjects was almost two-fold greater than that of the downregulated genes (**Figure 1A**). Furthermore, DESeq2 was used to screen differentially expressed miRNAs (DEmiRNAs) (**Figure 1B**). A total of 442 mRNAs and 11 miRNAs were screened by differential expression analysis. Most significantly upregulated miRNAs in metastasis PAAD samples were miR-934 and miR-6510. The target genes of DEmiRNAs were screened using R packages miRNAtap, multiMiR, and hoardeR (targetScan). Target genes and differentially expressed genes (DEGs) were then used to establish a miRNA-mRNA co-expression network (Cytoscape 3.6.1) (**Figure 1C**). The differentially expressed genes and miRNAs in miRNA-mRNA co-expression network were listed in **Supplementary Table 5**. Survival curve analysis of DEmiRNAs in the co-expression network (http://www.linkedomics.org/admin.php) is shown in **Figure 1D**. The expression of miRNA-934 was significantly associated with PAAD prognosis. Furthermore, the expression of miR-934 and miR-6510 in Hs766t cells and liver metastasis cell line Hs7667-L3 was verified by qRT-PCR (**Figure 1E**). miRNA-934 was significantly upregulated in Hs7667-L3 cells. Therefore, miR-934 was subjected to further analysis.

### Four-miRNA Panel Allowed for Risk Stratification and Survival Prediction in PAAD

**Figure 2:**
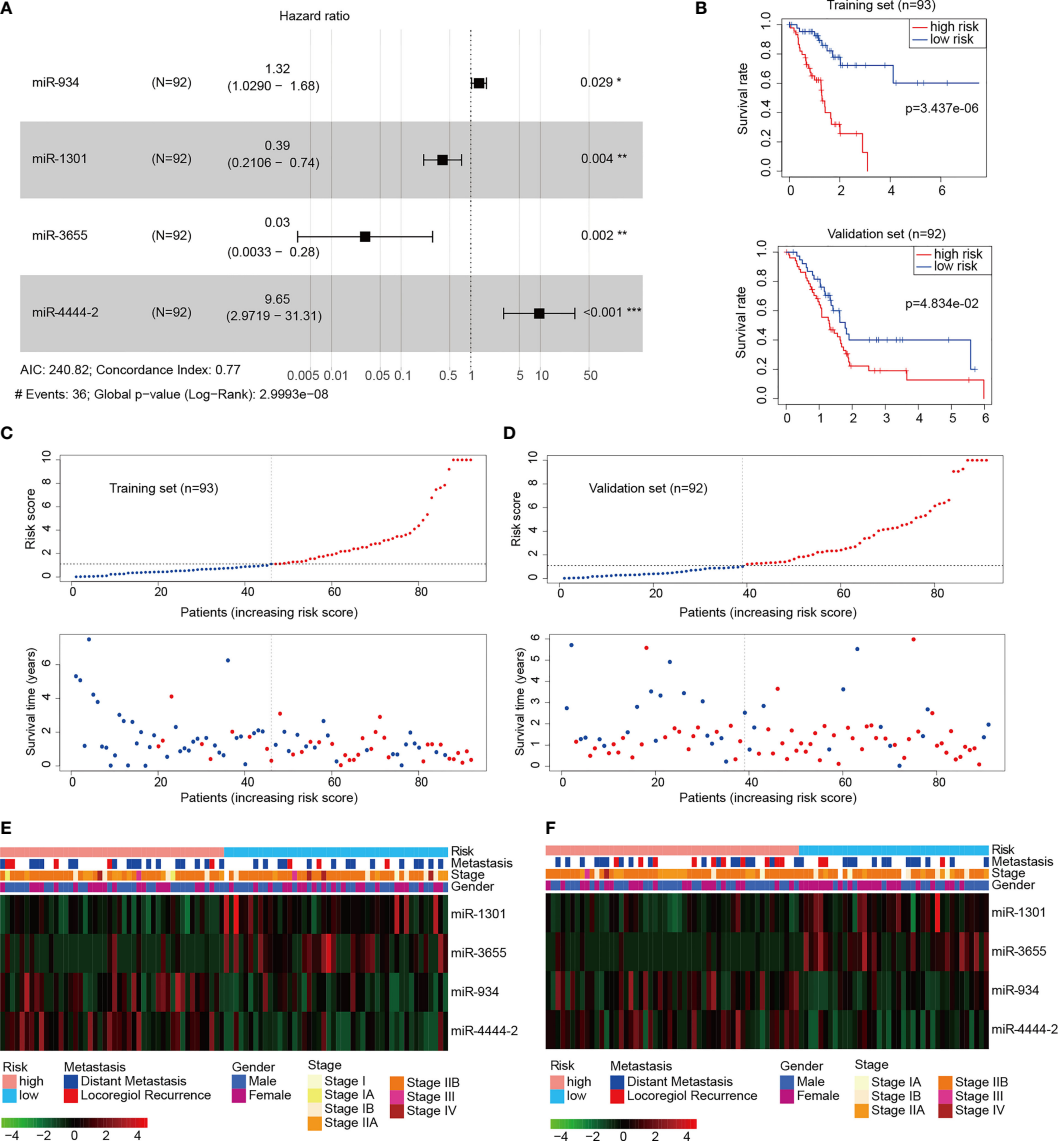
Construction of the pancreatic adenocarcinomas (PAAD) prognostic signature. **(A)** Forest plot of the 4-miRNA panel (miR-1301, miR-3655, miR-934, and miR-4444-2) multivariate Cox regression analysis in the validation cohort (n=92) from TCGA PAAD (Wald test, *P < 0.05, **P < 0.01, ***P < 0.001). **(B)** Kaplan-Meier curves of overall survival based on the high and low risk group in the training (n=93, top) and validation cohort (n=92, bottom) compared statistically using the log rank test. **(C, D)** Risk score distribution, survival status of each patient in the training cohort **(C)** and validation cohort **(D)**. **(E, F)** The heatmap plots of the 4-miRNA panel with characteristics including prognostic signature risk subgroup and clinical pathology in the training cohort **(E)** and validation cohort **(F)**.

To improve pancreatic cancer prognosis/survival prediction, the established 4-miRNA panel was screened *via* Lasso model computation based on miR-934. Panel miRNAs included miR-934, miR-3655, miR-1301, and miR-4444-2. The risk score = 0.274584* (the expression value of miR-934) + 2.266621* (the expression value of miR-4444-2) - 0.93094* (the expression value of miR-1301) - 3.49226 * (the expression value of miR-3655). The forest plot depicting hazard ratios and 95% confidence intervals of multivariate is presented in **Figure 2A**. miR-1301 and miR-3655 expression were significantly associated with better OS (Hazard ratio: 0.39; 95% confidence interval: 0.21-0.74; P=0.004 and Hazard ratio: 0.03; 95% confidence interval: 0.0033-0.28; P=0.002, respectively). Meanwhile, miR-934 (Hazard ratio: 1.32; 95% confidence interval: 1.029-1.68; P=0.029) and miR-4444-2 (Hazard ratio: 9.65; 95% confidence interval: 2.9719-31.31; P<0.001) showed significantly positive related to poor OS. To determine the clinical potential of our miRNA signature for the identification of high-risk PAAD patients, we examined whether the 4-miRNA panel could predict survival using Kaplan-Meier analysis. Training cohort analysis was presented in **Figure 2B** top plot, and validation cohort result was displayed in **Figure 2B** bottom plot. As expected, patients with high-risk scores had a significantly lower 5-year OS (~8%) compared to those in the low-risk group (~41%, p=3.437e-6) in validation cohort. According to the median threshold of risk score, a total of 93 PAAD patients in the training set were divided into high-risk and low-risk groups. As shown in **Figure 2C**, the death probability of PDDA patients augmented as the risk score increased. Besides, the heatmap visualized the expression pattern of those 4 miRNAs between the two risk subgroups. Similarly, in **Figure 2D**, based on the risk score, 92 PAAD patients in the validation cohort were also divided into high-risk and low-risk groups. To evaluate the relationship of the 4-miRNA panel with clinical characteristics, heatmap analysis was performed with risk score used to stratify patients into high- and low-risk groups (**Figures 2E, F**). Higher expression of miR-934 and miR-4444-2, as well as lower expression of miR-1301 and miR-3655 were observed in the high-risk group. The opposite expression patterns were observed in the low-risk group. Collectively, these results supported the clinical utility of our prognostic miRNA signature for PAAD patient stratification.

### Establishment of a Nomogram for Clinical Implementation of PAAD Risk Stratification

**Figure 3:**
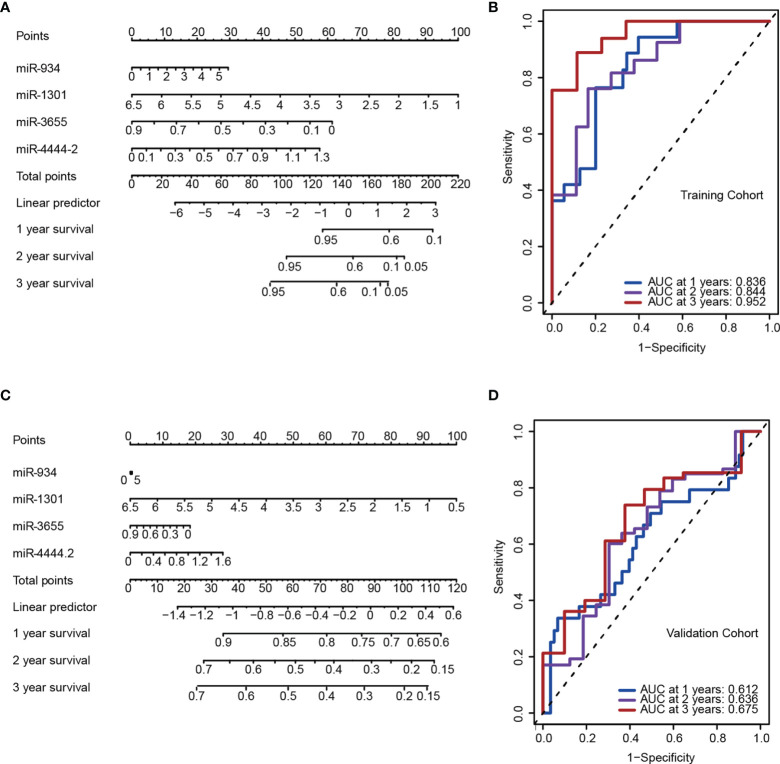
A 4-miRNA panel was established to predict the overall survival (OS) of pancreatic adenocarcinomas (PAAD) patients. **(A, C)** Nomogram model of the 4-miRNA panel for predicting the probability of 1-, 2-, and 3-year OS in the training **(A)** and the validation cohort **(C)**. **(B, D)** Time-dependent ROC analysis at 1, 2 and 3 years in the training **(B)** and the validation cohort **(D)**.

As an accurate quantitative method, the nomogram is widely used to assess the survival rate of patients. Using the factor scores in the nomogram, clinicians can obtain the predicted OS probability of individual patients and perform closer and precise treatment management of patients. We first constructed a nomogram containing the variables miRNA: miR-934, miR-4444-2, miR-1301, and miR-365 (**Figure 3**), and it was significantly associated with poor OS in predicting the probability of 1-, 2-, and 3-year OS in the PAAD training (**Figure 3A**) and validation cohort (**Figure 3B**). Furthermore, to validate the reliability of the 4 miRNAs signature in predicting prognosis through ROC analysis. The results showed that our risk model possessed the optimal predictive ability in the long-term survival rate for PAAD patients, with the AUC of 0.836, 0.844, and 0.952 in 1-, 2-, and 3-year, respectively (**Figures 3C, D**). The Kaplan-Meier analysis indicated that the OS was significantly different between the high- and low-risk groups of PAAD patients according to our risk model. All the above results proved the better prognostic value of 4 miRNAs signature.

### Nomogram Based on the Signature

**Figure 4:**
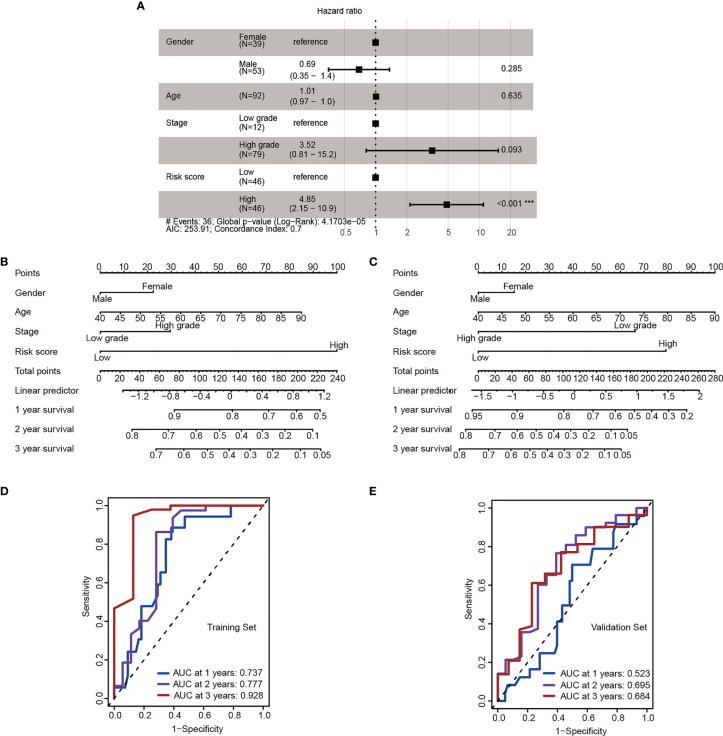
The 4-miRNA stratification signature is an independent risk factor. **(A)** Forest plot depicting hazard ratios and 95% confidence intervals of the univariate significant clinicopathological variables. Parameters colored in black are significant risk factors in multivariate analysis (Wald test, ***P < 0.001). **(B, C)** Nomogram derived from the combination of high- and low-risk group stratification and key clinicopathological parameters in the training **(B)** and the validation cohort **(C)**. **(D, E)** ROC for 1-, 2-, 3- year OS in the training **(D)** and the validation cohort **(E)**.

To further validation of independent prognosis value, the risk score model based on 4-miRNA was adjusted with several clinical characteristics. A forest plot demonstrated the association between gender, age, stage, risk score and overall survival was showed (**Figure 4A**). The nomogram was also established to illustrated the relationship between the signature (gender, age, stage, risk score) and the overall survival (**Figures 4B, C**). We found that the risk score model incorporated with traditional clinical indicators has a slight lower AUC than using the risk stratification itself, these results showed the risk stratification could be served as independent biomarker (**Figures 4D, E**).

### Enrichment Analysis Revealed Adhesion Molecule Binding Pathway Activation in the High-Risk Group

**Figure 5:**
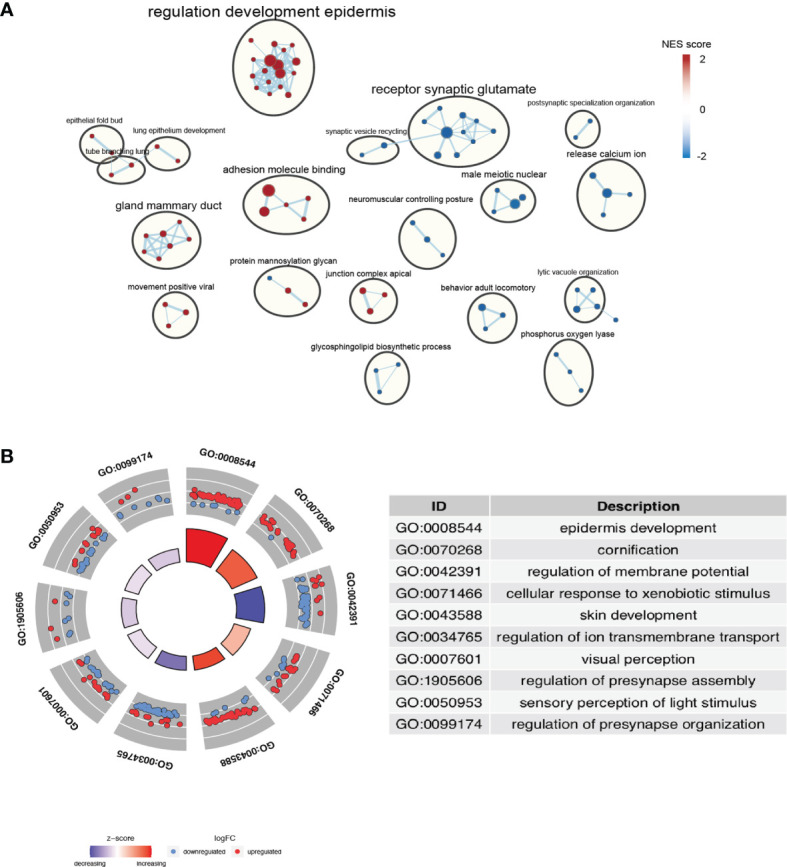
Identification of the pathways enriched of the high- and low- risk group. **(A)** Gene set enrichment analysis (GSEA) delineates KEGG pathways that characterize PAAD patients with high risk. **(B)** Enriched terms are colored by normalized enrichment score (NES), where nodes that share the same cluster ID are typically close to each other based on DEGs.

In order to explore the biological processes underlying the risk classification, GO and KEGG were used to analyze the main enrichment pathways in the high-risk and low-risk groups. The high-risk group was mainly enriched in signal pathways that bind to enveloped epidermis and adhesion molecules. Since the loss of tumor cell adhesion and dissociation *in situ* was early step of invasion and metastasis, these pathways activated in the high-risk group are positively correlated with the high invasiveness and metastasis of pancreatic cancer (**Figure 5**) (28). The pathways of “receptor synaptic glutamate” and “release calcium ion” were significantly downregulated in the high-risk group compared to those in the low-risk group. Increases in intracellular Ca^2+^ concentration have been suggested to promote cancer progression (29, 30). A decrease in Ca^2+^ transmembrane transport may facilitate a beneficial tumor microenvironment. Taken together, the increase in cell adhesion signaling and the decrease in Ca^2+^ release pathway was positively associated with the poor prognosis of high-risk group.

### Analysis of Immune Cell Proportions in High- and Low-Risk PAAD Groups

**Figure 6:**
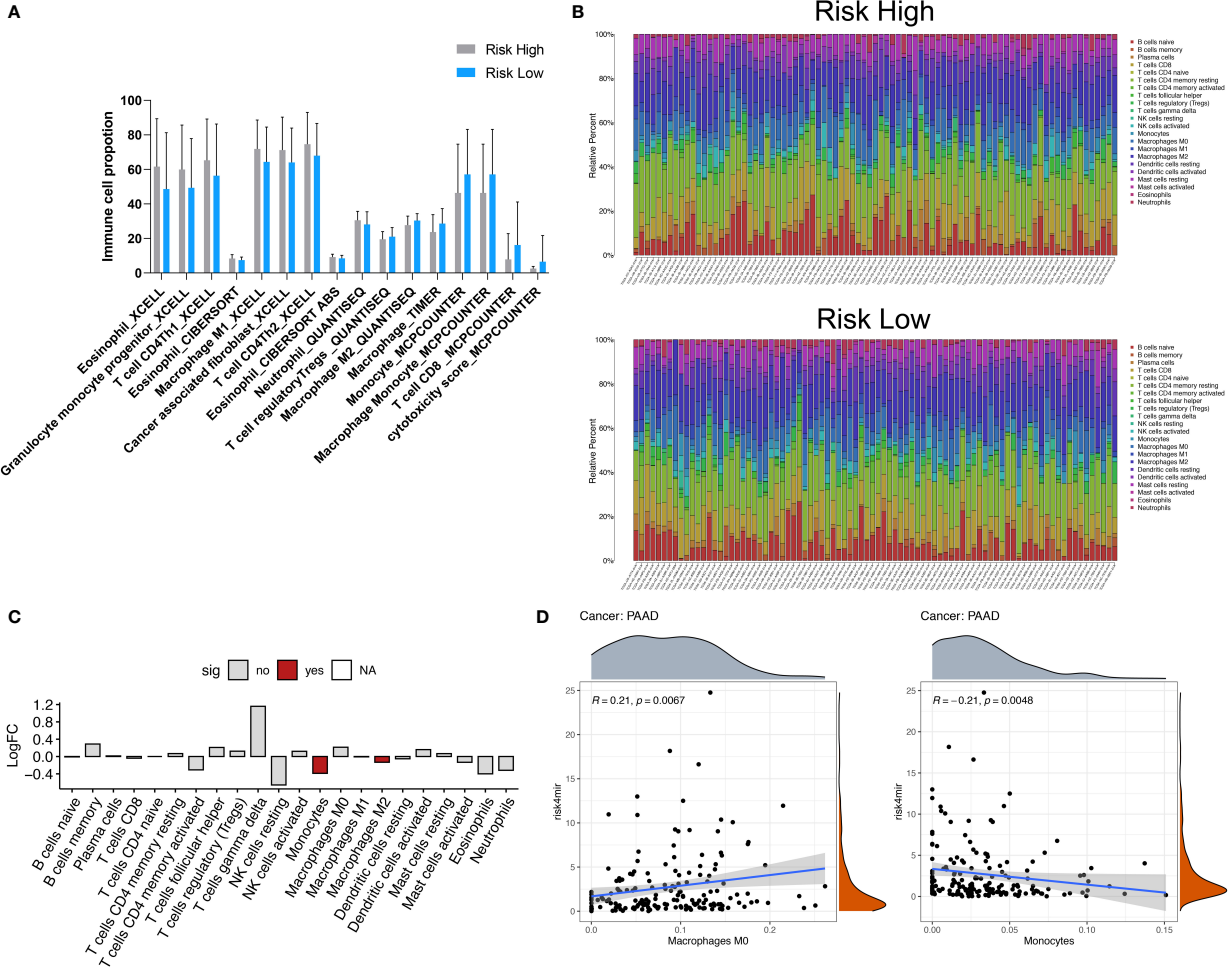
Immune cell type analyses correlated to 4 miRNA risk groups. **(A)** Comparison of the infiltrated immune cell levels between the high- and low-risk groups. The immune infiltrates scores based on CIBERSORT, XCELL, TIMER and MCPCOUNTER algorithms were retrieved from TIMER 2.0 database (http://timer.cistrome.org/). All the significant changed immune cell types was showed (Wilcoxon test, P < 0.05). **(B)** The abundance of 22 types of immune cells in TCGA PAAD based on CIBERSORT algorithms. **(C)** The significant differential immune cells types based on CIBERSORT algorithms. **(D)** The dot plot showed risk scores positively correlated with Macrophage M0 (R=0.21, P=0.0067) and negatively correlated with monocyte (R=-0.21, P=0.0048).

The occurrence of cancer is determined by the internal mutation of cancer cells and the external tumor microenvironment (TME). Immune cells in the tumor microenvironment regulate the growth and progression of tumors by producing a variety of pro-inflammatory cytokines (31, 32). The types, proportions and functional changes of tumor infiltrating immune cells will promote the occurrence and progression of PAAD. The immune infiltrates scores based on CIBERSORT, XCELL, TIMER and MCPCOUNTER algorithms were retrieved from TIMER 2.0 database (http://timer.cistrome.org/) indicated the comparison of the infiltrated immune cell levels between the high- and low-risk groups (**Figure 6A**), which shown the significant immune cell type proportions based on various algorithms, revealed that cancer associated fibroblast, CD4+ Th2 cells, M1 macrophages were upregulated in the high-risk group. In the low- risk group, CD8 T cells, monocytes, and cytotoxicity were upregulated. Moreover, the abundance of 22 types of immune cells in TCGA PAAD based on CIBERSORT algorithms was shown in **Figure 6B**. As we showed in **Figure 6C**, we found high risk groups have less Monocytes and Macrophage M2 infiltrated. The dot plot showed risk scores positively correlated with Macrophage M0 (R=0.21, p=0.0067) and negatively correlated with monocyte (R=-0.21, p=0.0048) (**Figure 6D**). These results may imply that bad prognosis in high-risk groups not *via* TME regulation, but through another kinds of mechanisms, maybe the pathway enriched like “bind to enveloped epidermis” and “adhesion molecules” have greater impact as we expected.

### Molecular Subtypes Classification of PAAD

**Figure 7:**
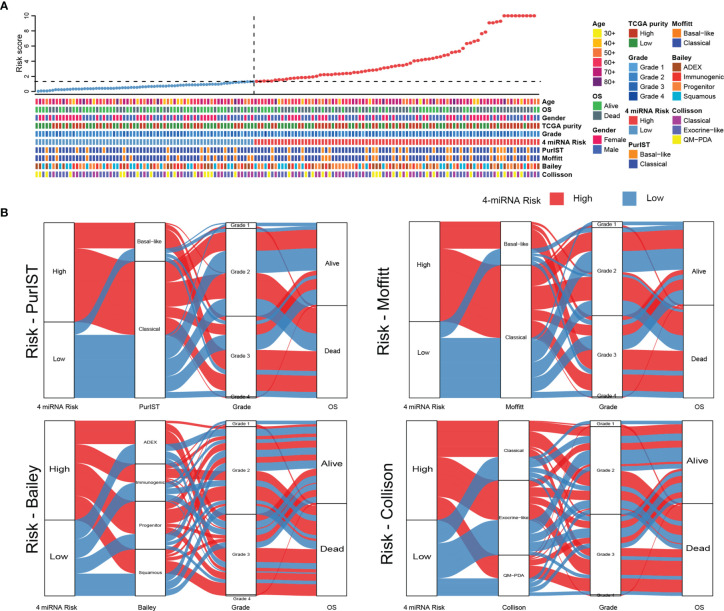
The comparison of 4-miRNA risk groups to molecular subtypes of PAAD. **(A)** The heat map showed the classification results with various molecular subtype system in TCGA PAAD cohort. The upper panel showed the samples were sorted by the 4-miRNA risk scores and the lower panel showed the detail subtype information. **(B)** The Sankey diagrams showed the relationship of high and low risk groups to each of molecular subtyping systems (PurIST, Moffitt, Bailey, Collison).

To further analysis the relationship of 4-miRNA risk groups with previous well-established molecular subtype systems, we retrieved information from the original categories data of Colisson, Bailey, Moffitt and PurIST (21–25). As showed in **Figure 7A**, the heat map showed our 4-miRNA risk groups was highly correlated to overall survival and showed a complex pattern related to the previous molecular subtype system (**Figures 7A, B**).

### The 4-miRNA Signature Was Validated in Two GSE Database

**Figure 8:**
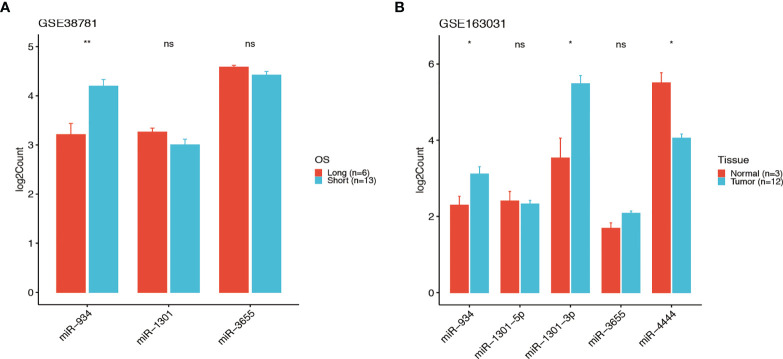
The signature of the 4-miRNA in two GSE database. The expressions of the 4 miRNAs in GEO database GSE38781 and GSE163031 (t test, ns P > 0.05, *P < 0.05, **P < 001). The higher expression of miR-934 and lower expression of miR-1301 and miR-3655 were predicted the poor prognosis in PDAC patients of GSE38781 **(A)**. The expressions of miR-934, miR-1301, miR-3655 and miR-4444 in PDAC patients of GSE163031 **(B)**.

GEO database GSE38781 and GSE163031 were used to reanalysis the relationship between the OS and the expression of the 4 miRNAs, we found the higher expression of miR-934 and lower expression of miR-1301 and miR-3655 were predicted the poor prognosis in pancreatic cancer (**Figures 8A, B**), which was consist with the 4-miRNA prognostic model we constructed in this study. Thus, these data support the potential of our miRNA signature in predict the survival of pancreatic cancer patients.

### Validation of the Predictive Ability of the Risk Model in a Clinical External Cohort

**Figure 9:**
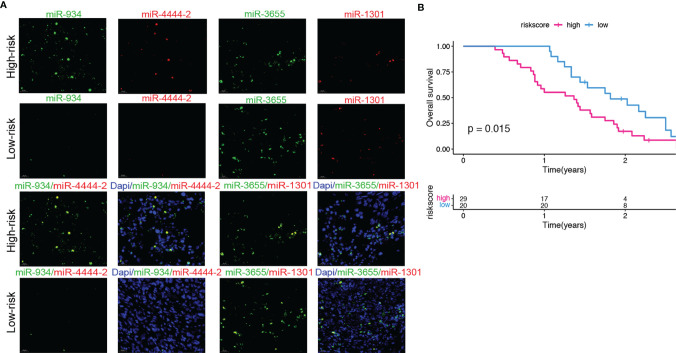
Validation of the predictive ability of the risk model in a clinical external cohort. **(A)** Representative images of FISH (40× magnification) of 4 miRNAs in PC patients tissue samples. **(B)** The correlation between overall survival based on clinical information and the scores of the risk group based on the 4 miRNAs expressions in PC patients tissue samples. Compared with the low-risk group, the high-risk group has a significantly lower overall survival rate. (log-rank test, P=0.015).

A clinical cohort that includes 90 pancreatic cancer (PC) patients with different clinical stages was constructed to verify the predictive ability of the risk score. Firstly, the expressions of 4 miRNAs of high and low risk model were remarkably different in various stages of PC through *in situ* hybridization analysis. We first scored the 4 miRNAs expressions in each pancreatic cancer patient based on the positive expression of miRNA. The score represents the expression of miRNA in the tissue sample (i.e. 25% positive expression rate = 1 point, 50% positive expression rate = 2 points, 75% positive expression rate = 3 points, 100 positive expression rate = 4 points). Secondly, we calculated the risk score based on the expressions of 4 miRNAs (**Supplementary Table 6**), and found high-risk groups (i.e. miR-934, miR-4444-2 high expression, miR-1301, miR-3655 low expression) survival prognosis was significantly (p<0.05) lower than the low-risk group (**Figure 9**). The results of this study confirmed the prognostic value of our 4-miRNA risk model.

### High-Risk Group Promoting Proliferation and Migration of PC Cells

**Figure 10:**
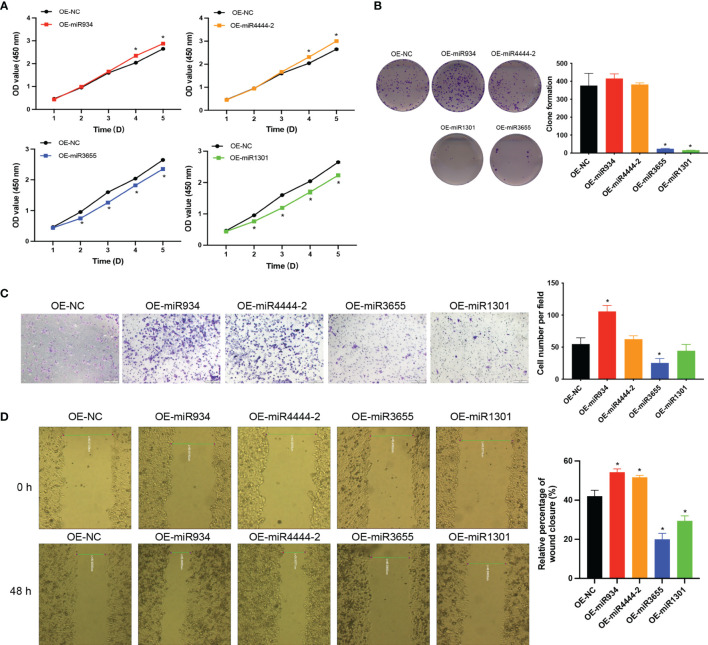
The effects of miR-934, miR-4444-2, miR-1301 and miR-3655 on proliferation and migration of PC cells. **(A, B)** Overexpressed of miR-934 and miR-4444-2 observably promoted the proliferation ability in PC cells compared with the NC group (Overexpression of miR-1301 and miR-3655 has the opposite effect on cells). **(C, D)** Overexpressed of miR-934 and miR-4444-2 could significantly increase the migration of PC cells (Overexpression of miR-1301 and miR-3655 has the opposite effect on cells) (t test, *P < 0.05).

To further explore the role of the 4 miRNAs in PC cells, we examined whether the overexpression of the 4 miRNAs could affect the proliferation and migration of CFPAC PC cells. As shown in **Figure 10**. The CCK-8 analysis revealed that the overexpressed of miR-934 and miR-4444-2 observably promoted the proliferation ability and overexpressed of miR-1301 and miR-3655 significantly suppressed the cell proliferation compared with the OE-NC group (**Figure 10A**). Meanwhile, the clone formation, transwell migration assay and wound healing assay were performed to investigate the promoting effects of overexpressed miR-934 and miR-4444-2, and the inhibiting effects of overexpressed miR-1301 and miR-3655. The results demonstrated that the bad prognosis in high-risk group may due to significantly difference in the proliferation and migration of CFPAC PC cells when compared with low-risk group (**Figures 10B–D**).

## Discussion

Pancreatic cancer has occult metastasis and heterogeneity, but the current pathological staging system is completely based on the anatomical characteristics of the tumor and does not consider the tumor heterogeneity, which affects the prognosis of PAAD (33). To improve prognosis and personalized treatment, researchers previously proposed prognostic marker models for pancreatic cancer (34–36). In this study, we established and validated a 4-miRNA prognosis panel by using TCGA PAAD and GEO cohorts (GSE38781 and GSE163031). To further validate the advantages of 4-miRNA panel, we compared it to the four above-described models. The signature established in the current study was superior to those previously reported. Our 4-miRNA panel exhibited superior prognostic performance with excellent AUC scores of 0.836, 0.844, and 0.952 for the prediction of 1-, 2-, and 3- year survival. We then established a nomogram, which combined multiple independent prognostic variables. The nomogram robustly predicted risk and OS based on clinical stage, age, and other factors, in addition to miRNA expression. Taken together, the predictive model developed herein will improve the clinical management of PAAD, allowing for more personalized treatment choices.

Studies have reported on a risk scoring system based on prognostic-related miRNA, and significantly related miRNAs can be used as potential prognostic biomarkers of PAAD for the prognostic model of pancreatic cancer (37–40). In this study, we found that 4 miRNAs are significantly related to the prognosis of PAAD, and they were verified by GEO database. Among all the verified samples, high expression of miR-934 was consistently found to be significantly associated with poor prognosis of pancreatic cancer and several studies have shown that miR-934 promotes cancer metastasis (41–48). Related, this miRNA has not been reported in PAAD, but an article reported that miR-934 is significantly up-regulated in pancreatic ductal adenocarcinoma (PDAC) and is included in the 9-miRNA panel to predict poor survival (45). At the same time, miR-934 acts as an oncogene in ovarian cancer (46, 47). Recently, a study reported that miR-934, a prognostic marker, promotes cell proliferation and migration of pancreatic tumors by targeting PROX1 (48). Another up-regulated miRNA is rarely reported, miRNA microarray analysis showed that miR-4444 (FC>2) was significantly up-regulated in colorectal cancer and promoted tumor growth (49). This is consistent with the results of our study, that is, the up-regulation of miR-934 and miR-4444-2 was detected in the high-risk group of PAAD. On the other hand, studies have shown that the down-regulation of miR-1301 is associated with the malignant clinical features and poor OS of patients with colorectal cancer (50), and the overexpression of miR-1301 has been shown to inhibit cell proliferation, migration and invasion (51). It is worth noting that miR-1301-3p has been widely studied in liver cancer, breast cancer and other cancer types for its anti-tumor effect (52–55). Regarding miR-3655, wu et al. showed that miR-3655 down-regulates CXCL5 and is used as a new diagnostic and prognostic marker for osteosarcoma (56). Thus, miR-1301 and miR-3655 as the cancer suppressor genes negatively correlated with PAAD progression in our study, that was consistent with previous reports. However, there was no integrally study investigated miR-934, miR-4444-2, miR-1301 and miR-3655 in PAAD to date. Our study was start with a miRNA screening based on metastatic and non-metastatic PAAD database, that indicated a prognostic marker of advanced PAAD patients was identified. Based on this strategy, we found that miR-934 up regulated was positively associated with PAAD metastasis, further a significantly high expression of miR-934 was confirmed in PAAD lung metastasis cell line Hs766T-L3. Further, by Lasso method, there was a high- and low- risk group determined by the expression of miR-934, miR-4444-2, miR-1301 and miR-3655 was established, and this 4-miRNA panel was extremely correlated with the prognosis of PAAD. This study not only combined previously discovered miRNAs related to prognosis, but also found that their expression can effectively predict the prognosis of PAAD.

Recently, it has been reported that tumor immune subtypes are based on the interaction between tumor cells and immune cells. Patients belonging to the “immune escape” subtype usually have a poor prognosis (31, 32). Although we cannot directly assess the utility of our miRNA panel on the proposed tumor immune subtypes, it is exciting to observe that the panel-based risk stratification is related to the proportion of immune cells. Studies have shown that inflammatory M1 macrophages of pancreatic cancer are stimulated by KrasG12D acinar cells, and release factors such as NF-κB and Notch that lead to improper activation of signal pathways to stimulate acinar to ductal metaplasia (ADM) and then undergo carcinogenesis (31, 32). At the same time, unlike M1, M2 macrophages play a leading role in chronic pancreatitis fibrosis and β cell proliferation (57). Thus, there would be a high risk in an increasing proportion of M1 macrophages cells in PAAD.

The external clinical sample validation indicated that the 4 miRNAs were differentially expressed in patients in the high- and low-risk groups, and miR-934 and miR-4444-2 were elevated in high-risk PC tissues. miR-934 has been proven could promote tumor progression in several cancer types, such as ovarian and prostate cancer, but the role of miR-4444-2, miR-1301 and miR-3655 in PC is rarely studied. Therefore, we verified the effect of the 4 miRNAs in PC cells, and the results confirmed the promoting effects of miR-934 and miR-4444-2, the inhibiting effects of miR-1301 and miR-3655 in PC cell proliferation and migration. These results were consistent with the results in other cancers types. The target genes of miR-934, miR-4444-2, miR-1301 and miR-3655 were predicted and analyzed by the miRNA target gene prediction website (miRDB: http://mirdb.org/cgi-bin/search.cgi; Targent Scan Human: http://www.targetscan.org/vert_72/; miRWalk: http://www.targetscan.org) (**Supplementary Table 7**). We found that miR-934 promotes EMT could through PTEN-mediated PI3K pathway and AKT pathway, and the high expression of miR-4444-2 may promote the proliferation and migration of tumor cells by inhibiting the expression of the transcription factor E2F1, but more experiments are needed to explain and prove. Here, we focus on introducing an excellent high-risk prediction model for pancreatic cancer.

## Conclusion

In the current study, we successfully established a reliable risk model with multiple miRNAs in PAAD prognostic prediction, which was constituted by a signature of 4 miRNAs, as important risk assessment biomarkers. The risk nomogram developed herein can be used to accurately predict outcomes in PAAD patients. Implementing this nomogram-based risk stratification in the clinic can assist clinicians in identifying patients belonging to high-risk groups and choosing optimal treatment.

## Limitations

This study still has some limitations. One limitation is that the data we used to construct the risk stratification model comes from public databases TCGA, although we used some clinically pancreatic cancer tissue samples to validate the model, the samples came from one single center and the number is small. Further validation by combining multiple centers data was essential. Secondly, few works were done for exploring the molecular mechanism underlying the bad prognosis in high-risk group, we are currently doing more work to track and study the changes in the tumor and its microenvironment subjected to expression manipulating of each of four miRNAs.

## Data Availability Statement

The original contributions presented in the study are included in the article/**Supplementary Material**. Further inquiries can be directed to the corresponding authors.

## Author Contributions

Data analysis: XG and YL. Funding acquisition: XL. Methodology: PT and MP. Project administration: XL and YL. Resources: YP. Software: YL and YP. Supervision: XG and YL. Validation: CZ. Writing – original draft: XG. Writing – review & editing: YL. All authors contributed to the article and approved the submitted version.

## Funding

This work was supported by the National Natural Science Foundation of China (grant numbers 81430063), Guangdong Provincial Science and Technology Program (grant numbers 2019B030301009), Natural Science Foundation of Guangdong Province of China (grant numbers 2021A1515012161), Guangdong Province Regional Joint Fund-Key Projects (grant numbers 2020B1515120096), Sanming Project of Medicine in Shenzhen (grant numbers SZSM202003009), Shenzhen Key Laboratory Foundation (grant numbers ZDSYS20200811143757022) and Shenzhen International Cooperative Research Project (grant numbers GJHZ20200731095210030).

## Conflict of Interest

The authors declare that the research was conducted in the absence of any commercial or financial relationships that could be construed as a potential conflict of interest.

## Publisher’s Note

All claims expressed in this article are solely those of the authors and do not necessarily represent those of their affiliated organizations, or those of the publisher, the editors and the reviewers. Any product that may be evaluated in this article, or claim that may be made by its manufacturer, is not guaranteed or endorsed by the publisher.
