# Supplementary material for: Establishment of a 4-miRNA Prognostic Model for Risk Stratification of Patients With Pancreatic Adenocarcinoma
Source: Front Oncol. 2022 Feb 3;12:827259. doi: 10.3389/fonc.2022.827259 (PMC8851918; doi:10.3389/fonc.2022.827259)
Supplement: Supplementary file 1 [file DataSheet_1.pdf]

| | Level | High | Low | *p* |
|---|---|---|---|---|
| ***n*** | | 52 | 39 | |
| **Age at initial pathologic diagnosis (mean (SD))** | | 64.63 (11.35) | 67.00 (11.02) | 0.322 |
| **gender (%)** | Female | 21 (40.4) | 22 (56.4) | 0.193 |
| | Male | 31 (59.6) | 17 (43.6) | |
| **race (%)** | Not Evaluated | 0 (0.0) | 1 (2.6) | 0.58 |
| | Asian | 5 (9.6) | 3 (7.7) | |
| | Black or African American | 3 (5.8) | 1 (2.6) | |
| | White | 44 (84.6) | 34 (87.2) | |
| **Ajcc pathologic tumor stage (%)** | Discrepancy | 0 (0.0) | 2 (5.1) | 0.346 |
| | Stage IA | 1 (1.9) | 2 (5.1) | |
| | Stage IB | 2 (3.8) | 4 (10.3) | |
| | Stage IIA | 11 (21.2) | 6 (15.4) | |
| | Stage IIB | 36 (69.2) | 25 (64.1) | |
| | Stage III | 1 (1.9) | 0 (0.0) | |
| | Stage IV | 1 (1.9) | 0 (0.0) | |
| **Clinical stage (%)** | | 52 (100.0) | 39 (100.0) | NA |
| **Histological type (%)** | Discrepancy | 0 (0.0) | 1 (2.6) | 0.448 |

| | | | | |
|---|---|---|---|---|
| | Pancreas-Adenocarcinoma-Other Subtype | 4 (7.7) | 5 (12.8) | |
| | Pancreas-Adenocarcinoma Ductal Type | 46 (88.5) | 30 (76.9) | |
| | Pancreas-Colloid (mucinous non-cystic) Carcinoma | 2 (3.8) | 2 (5.1) | |
| | Pancreas-Undifferentiated Carcinoma | 0 (0.0) | 1 (2.6) | |
| **Histological grade (%)** | G1 | 6 (11.5) | 5 (12.8) | 0.184 |
| | G2 | 35 (67.3) | 18 (46.2) | |
| | G3 | 10 (19.2) | 13 (33.3) | |
| | G4 | 0 (0.0) | 2 (5.1) | |
| | GX | 1 (1.9) | 1 (2.6) | |
| **Initial pathologic dx year (mean (SD))** | | 2011.12 (1.60) | 2011.08 (2.15) | 0.927 |
| **Menopause status (%)** | | 52 (100.0) | 39 (100.0) | NA |
| **Birth days to (mean (SD))** | | -23804.62 (4158.43) | -24650.90 (4017.67) | 0.332 |
| **Vital status (%)** | Alive | 13 (25.0) | 19 (48.7) | 0.034 |
| | Dead | 39 (75.0) | 20 (51.3) | |
| **Tumor status (%)** | | 6 (11.5) | 4 (10.3) | 0.2 |
| | Tumor free | 10 (19.2) | 14 (35.9) | |

| | | | | |
|---|---|---|---|---|
| | With tumor | 36 (69.2) | 21 (53.8) | |
| **Last contact days to (mean (SD))** | | 720.08 (526.21) | 840.05 (526.66) | 0.531 |
| **Death days to (mean (SD))** | | 462.00 (381.92) | 492.45 (404.02) | 0.777 |
| **Cause of death (%)** | | 21 (40.4) | 21 (53.8) | 0.731 |
| | Other malignancy | 1 (1.9) | 1 (2.6) | |
| | Other, non-malignant disease | 2 (3.8) | 2 (5.1) | |
| | Other, specify | 1 (1.9) | 0 (0.0) | |
| | Pancreatic Cancer | 26 (50.0) | 14 (35.9) | |
| | Surgical Complications | 1 (1.9) | 1 (2.6) | |
| **New tumor event type (%)** | | 24 (46.2) | 23 (59.0) | 0.298 |
| | Distant Metastasis | 17 (32.7) | 12 (30.8) | |
| | Locoregional Recurrence | 10 (19.2) | 3 (7.7) | |
| | Locoregional Recurrence\|Distant Metastasis | 0 (0.0) | 1 (2.6) | |
| | New Primary Tumor | 1 (1.9) | 0 (0.0) | |
| **New tumor event site (%)** | | 28 (53.8) | 23 (59.0) | 0.604 |
| | Liver | 8 (15.4) | 8 (20.5) | |
| | Lung | 3 (5.8) | 3 (7.7) | |

| | | | | |
|---|---|---|---|---|
| | Other, specify | 9 (17.3) | 4 (10.3) | |
| | Peritoneal Surfaces | 3 (5.8) | 0 (0.0) | |
| | Tumor Bed | 1 (1.9) | 1 (2.6) | |
| **New tumor event site other (%)** | | 44 (84.6) | 35 (89.7) | 0.495 |
| | Adrenal and right buttock | 1 (1.9) | 0 (0.0) | |
| | bone | 0 (0.0) | 1 (2.6) | |
| | Liver/lung | 1 (1.9) | 0 (0.0) | |
| | Lung and Liver | 1 (1.9) | 0 (0.0) | |
| | malignant ascites | 1 (1.9) | 0 (0.0) | |
| | Omentum | 1 (1.9) | 0 (0.0) | |
| | Omentum, Peritoneal surface, Non regional Lymph nodes | 1 (1.9) | 0 (0.0) | |
| | perigastric mass | 0 (0.0) | 1 (2.6) | |
| | Regional LNs | 1 (1.9) | 0 (0.0) | |
| | retroperitoneal recurrence around the supramesenteric artery and vein and some para aortic nodes | 0 (0.0) | 1 (2.6) | |
| | Retroperitoneum | 1 (1.9) | 0 (0.0) | |
| | Superior Mesenteric Artery | 0 (0.0) | 1 (2.6) | |

| | | | | |
|---|---|---|---|---|
| **New tumor event dx days to (mean (SD))** | | 380.25 (352.88) | 478.88 (391.54) | 0.396 |
| **Treatment outcome first course (%)** | | 1 (1.9) | 1 (2.6) | 0.866 |
| | Discrepancy | 2 (3.8) | 1 (2.6) | |
| | Unknown | 5 (9.6) | 6 (15.4) | |
| | Complete Remission/Response | 20 (38.5) | 17 (43.6) | |
| | Partial Remission/Response | 3 (5.8) | 1 (2.6) | |
| | Progressive Disease | 17 (32.7) | 12 (30.8) | |
| | Stable Disease | 4 (7.7) | 1 (2.6) | |
| **Margin status (%)** | | 52 (100.0) | 39 (100.0) | NA |
| **Residual tumor (%)** | | 52 (100.0) | 39 (100.0) | NA |
| **OS (mean (SD))** | | 0.75 (0.44) | 0.51 (0.51) | 0.019 |
| **OS.time (mean (SD))** | | 526.52 (431.93) | 661.79 (493.94) | 0.168 |
| **DSS (mean (SD))** | | 0.63 (0.49) | 0.39 (0.50) | 0.027 |
| **DSS.time (mean (SD))** | | 526.52 (431.93) | 661.79 (493.94) | 0.168 |
| **DFI (mean (SD))** | | 0.53 (0.51) | 0.18 (0.39) | 0.029 |
| **DFI.time (mean (SD))** | | 468.74 (446.80) | 859.06 (592.80) | 0.031 |
| **PFI (mean (SD))** | | 0.75 (0.44) | 0.56 (0.50) | 0.063 |
| **PFI.time (mean (SD))** | | 418.31 (380.03) | 557.41 (484.59) | 0.128 |

| | | | | |
|---|---|---|---|---|
| **Redaction (%)** | | 52 (100.0) | 39 (100.0) | NA |
| **Risk (%)** | High | 52 (100.0) | 0 (0.0) | <0.001 |
| | Low | 0 (0.0) | 39 (100.0) | |
