# Supplementary material for: Establishment of a 4-miRNA Prognostic Model for Risk Stratification of Patients With Pancreatic Adenocarcinoma
Source: Front Oncol. 2022 Feb 3;12:827259. doi: 10.3389/fonc.2022.827259 (PMC8851918; doi:10.3389/fonc.2022.827259)
Supplement: Supplementary file 2 [file DataSheet_2.pdf]

	Level	High	Low	*p*
n		46	46	
**Age at initial pathologic diagnosis (mean (SD))**		64.96 (10.28)	62.11 (11.16)	0.206
**gender (%)**	Female	19 (41.3)	20 (43.5)	1
	Male	27 (58.7)	26 (56.5)	
**race (%)**	Unknown	2 (4.3)	1 (2.2)	0.201
	Asian	4 (8.7)	0 (0.0)	
	Black or African American	1 (2.2)	1 (2.2)	
	White	39 (84.8)	44 (95.7)	
**Ajcc pathologic tumor stage (%)**		0 (0.0)	1 (2.2)	0.487
	Stage I	0 (0.0)	1 (2.2)	
	Stage IA	2 (4.3)	0 (0.0)	
	Stage IB	4 (8.7)	5 (10.9)	
	Stage IIA	8 (17.4)	4 (8.7)	
	Stage IIB	30 (65.2)	31 (67.4)	
	Stage III	1 (2.2)	1 (2.2)	
	Stage IV	1 (2.2)	3 (6.5)	
**Clinical stage (%)**		46 (100.0)	46 (100.0)	NA

**Histological type (%)**	Pancreas-Adenocarcinoma-Other Subtype	6 (13.0)	12 (26.1)	0.189
	Pancreas-Adenocarcinoma Ductal Type	40 (87.0)	34 (73.9)	
**Histological grade (%)**	G1	6 (13.0)	14 (30.4)	0.019
	G2	21 (45.7)	24 (52.2)	
	G3	19 (41.3)	8 (17.4)	
**Initial pathologic dx year (mean (SD))**		2011.48 (1.59)	2011.28 (1.77)	0.578
**Menopause status (%)**		46 (100.0)	46 (100.0)	NA
**Birth days to (mean (SD))**		-23919.98 (3740.05)	-22891.85 (4064.92)	0.21
**Vital status (%)**	Alive	19 (41.3)	37 (80.4)	<0.001
	Dead	27 (58.7)	9 (19.6)	
**Tumor status (%)**		6 (13.0)	4 (8.7)	0.563
	Discrepancy	0 (0.0)	1 (2.2)	
	Tumor free	16 (34.8)	20 (43.5)	
	With tumor	24 (52.2)	21 (45.7)	
**Last contact days to (mean (SD))**		428.68 (237.96)	722.46 (639.38)	0.059
**Death days to (mean (SD))**		370.78 (280.57)	547.11 (412.62)	0.157

**Cause of death (%)**		28 (60.9)	37 (80.4)	0.157
	Other, non-malignant disease	1 (2.2)	0 (0.0)	
	Pancreatic Cancer	16 (34.8)	9 (19.6)	
	Surgical Complications	1 (2.2)	0 (0.0)	
**New tumor event type (%)**		23 (50.0)	29 (63.0)	0.444
	Distant Metastasis	17 (37.0)	13 (28.3)	
	Locoregional Recurrence	5 (10.9)	3 (6.5)	
	Locoregional Recurrence|Distant Metastasis	0 (0.0)	1 (2.2)	
	New Primary Tumor	1 (2.2)	0 (0.0)	
**New tumor event site (%)**		23 (50.0)	31 (67.4)	0.551
	Liver	12 (26.1)	9 (19.6)	
	Lung	2 (4.3)	2 (4.3)	
	Non-regional / Distant Lymph Nodes	2 (4.3)	0 (0.0)	
	Other, specify	3 (6.5)	1 (2.2)	
	Peritoneal Surfaces	2 (4.3)	2 (4.3)	
	Tumor Bed	2 (4.3)	1 (2.2)	
**New tumor event site other (%)**		43 (93.5)	44 (95.7)	0.414
	Left shoulder	1 (2.2)	0 (0.0)	

	Liver	0 (0.0)	1 (2.2)	
	Omentum	1 (2.2)	0 (0.0)	
	Subcut nodule	1 (2.2)	0 (0.0)	
	Tumor Bed and Liver	0 (0.0)	1 (2.2)	
**New tumor event dx days to (mean (SD))**		252.26 (164.27)	497.82 (251.76)	0.001
**Treatment outcome first course (%)**		2 (4.3)	2 (4.3)	0.176
	Discrepancy	2 (4.3)	1 (2.2)	
	Unknown	6 (13.0)	12 (26.1)	
	Complete Remission/Response	17 (37.0)	19 (41.3)	
	Partial Remission/Response	6 (13.0)	0 (0.0)	
	Progressive Disease	12 (26.1)	10 (21.7)	
	Stable Disease	1 (2.2)	2 (4.3)	
**Margin status (%)**		46 (100.0)	46 (100.0)	NA
**Residual tumor (%)**		46 (100.0)	46 (100.0)	NA
**OS (mean (SD))**		0.59 (0.50)	0.20 (0.40)	<0.001
**OS.time (mean (SD))**		394.70 (262.61)	688.15 (601.88)	0.003
**DSS (mean (SD))**		0.43 (0.50)	0.20 (0.40)	0.015
**DSS.time (mean (SD))**		394.70 (262.61)	688.15 (601.88)	0.003

**DFI (mean (SD))**		0.24 (0.44)	0.37 (0.50)	0.401
**DFI.time (mean (SD))**		312.94 (269.80)	703.37 (416.11)	0.002
**PFI (mean (SD))**		0.59 (0.50)	0.41 (0.50)	0.097
**PFI.time (mean (SD))**		302.50 (249.66)	611.67 (547.00)	0.001
**Redaction (%)**		46 (100.0)	46 (100.0)	NA
**Risk (%)**	High	46 (100.0)	0 (0.0)	<0.001
	Low	0 (0.0)	46 (100.0)	
