# Supplementary material for: Establishment of a 4-miRNA Prognostic Model for Risk Stratification of Patients With Pancreatic Adenocarcinoma
Source: Front Oncol. 2022 Feb 3;12:827259. doi: 10.3389/fonc.2022.827259 (PMC8851918; doi:10.3389/fonc.2022.827259)
Supplement: Supplementary file 3 [file Table_1.docx]

| **Supplementary Table 3. Sequences of miRNAs qRT-PCR primers** | | | |
| --- | --- | --- | --- |
| Species | miRNA | Forward (5′-3′) | Reverse (5′-3′) |
| Human | miRNA-934 | CGATGCTGATGTCTACTACTGGA | TATGGTTGTTCTGCTCTCTGTCTC |
| Human | miRNA-6510 | TTCTCGCTCTCGAGTTGGAAG | TATGGTTGTTCACGACTCCTTCAC |
| Human | U6-control | CGCTTCGGCAGCACATATAC | TTCACGAATTTGCGTGTCATC |
