# Supplementary material for: Establishment of a 4-miRNA Prognostic Model for Risk Stratification of Patients With Pancreatic Adenocarcinoma
Source: Front Oncol. 2022 Feb 3;12:827259. doi: 10.3389/fonc.2022.827259 (PMC8851918; doi:10.3389/fonc.2022.827259)
Supplement: Supplementary file 4 [file Table_2.docx]

| **Supplementary Table 4. Sequences of the 4 miRNAs mimics** | | | |
| --- | --- | --- | --- |
| Species | miRNA | 5′-3′ | 5′-3′ |
| Human | miRNA-1301 | UUGCAGCUGCCUGGGAGUGACUUC | AGUCACUCCCAGGCAGCUGCAAUU |
| Human | miRNA-3655 | GCUUGUCGCUGCGGUGUUGCU | CAACACCGCAGCGACAAGCUU |
| Human | miRNA-934 | UGUCUACUACUGGAGACACUGG | AGUGUCUCCAGUAGUAGACAUU |
| Human | miRNA-4444.2 | CUCGAGUUGGAAGAGGCG | CCUCUUCCAACUCGAGUU |
