# Supplementary material for: Establishment of a 4-miRNA Prognostic Model for Risk Stratification of Patients With Pancreatic Adenocarcinoma
Source: Front Oncol. 2022 Feb 3;12:827259. doi: 10.3389/fonc.2022.827259 (PMC8851918; doi:10.3389/fonc.2022.827259)
Supplement: Supplementary file 8 [file Table_6.docx]

Abbreviations list

| PAADs | Pancreatic Adenocarcinomas |
| --- | --- |
| PC | Pancreatic Cancer |
| PDAC | Pancreatic Ductal Adenocarcinoma |
| TCGA | The Cancer Genome Atlas |
| HCMDB | Human Cancer Metastasis Database |
| LASSO | Least Absolute Shrinkage and Selection Operator |
| OS | Overall Survival |
| AUC | Area Under Curve |
| ROC | Receiver Operating Characteristic |
| CCK-8 | Cell Counting Kit-8 |
| CA199 | Carbohydrate Antigen199 |
| GEO | Gene Expression Omnibus |
| TNM | Tumor Node Metastasis |
| CPM | Count-Per-Millon |
| FC | Fold Change |
| GO | Gene Ontology |
| KEGG | Kyoto Encyclopedia of Genes and Genomes |
| GSEA | Gene Set Enrichment Analysis |
| FDR | False Discovery Rate |
| qRT-PCR | Quantitative Real-time PCR |
| FISH | Fluorescence in situ hybridization |
| DAPI | 4',6-diamidino-2-phenylindole |
| DMEM | Dulbecco’s modified Eagle’s medium |
| FBS | Fetal Bovine Serum |
| OD | Optional Density |
| PBS | Phosphate Buffered Saline |
| SD | Standard Deviation |
| DEmiRNAs | Differentially Expressed miRNAs |
| DEGs | Differentially Expressed Genes |
| TME | Tumor Microenvironment |
| CD4+ Th2 | Cluster of Differentiation 4+ T helper 2 cell |
| CD8 | Cluster of Differentiation 8 |
| OE-NC | Over Expression-Negative Control |
| PROX1 | Homeobox prospero-like protein 1? |
| NF-κB | Nuclear Factor kappa-B |
| ADM | Acinar to Ductal Metaplasia |
| EMT | Epithelial-Mesenchymal Transition |
| PTEN | Phosphatase and tensin homolog deleted on chromosome ten |
| PI3K | Phosphatidylinositide 3-kinases |
| E2F1 | E2F transcription factor 1 |
